# Modern Scepticism
*Letters on the Laws of Man's Nature and Development. By Henry George Atkinson, F.G.S., and Harriet Martineau. London: Chapman.


**Published:** 1851-07-01

**Authors:** 


### THE JOURNAL

OF

### PSYCHOLOGICAL MEDICINE

AND

### MENTAL PATHOLOGY.

JULY 1, 1851.

### Art. I.—MODERN SCEPTICISM.*

With an extraordinary degree of feminine simplicity, a lady thus addresses a gentleman: "I want you to tell me with great particularity, (if you will,) how you would have one set about the study of the powers of man, in order to understand his nature, and his place, business, and pleasure, in the universe."

We remember hearing that Dr. Darwin and Miss Seward were for some period engaged, with the aid of a pestle and mortar, in certain chemical manipulations in his study at Lichfield. Of the product of this subtle alchymy, history has left no record; but, from the character of the parties, we have no doubt that it was far more innocent than the spells of Manfred and Astarte. The philosophical union of this "Benthamite spinster" and this "geological fellow," however, must be still more so; scandal cannot cast even a suspicion on the purity of their Platonism, indulged as it was through the prudent medium of epistolary correspondence.

The response of the gentleman is equally free and open; the offer is accepted as soon as made, and he acquiesces joyfully in the lady's proposition, and proposes to give her a notion how he *"came by his scientific basis."* It will be our object to follow in the wake of this pair of learned Thebans, and to wander with them through the mazes of their Platonic reveries. The contract is between two reflective but misguided minds, and the intellectual marriage is consummated with great skill and energy; but we shall see whether they are justified in

* Letters on the Laws of Man's Nature and Development. By Henry George Atkinson, F.G.S., and Harriet Martineau. London: Chapman.

palming their offspring on the world in order to arraign this scientific age for its ignorance.

To Mr. Atkinson she exclaims, "it is strange to think how many books I have read, and what an amount of hours I have spent in thinking, without being ever for one moment satisfied that I knew what I was about." We are not astonished at this result, when we hear her own account of the course she pursued. If there be any one mode more certain than another to make a chaos of our reasoning faculties, it is to ponder over the reveries of metaphysical dreamers, from Plato downwards; to attempt to analyze the annals of pseudo-psychology, from Pyrrho to Paley.

We think that such a course of reading would as soon render the mind "like sweet bells jangled and out of tune," as the pseudomenos, or the riddle of the sphinx, the longitude, or perpetual motion.

Yet we think that the lady, in her course of study, might have stumbled on the truth, that there are *some* mental philosophers who have been long engaged in "an experimental inquiry into the science of mind," instead of wondering where to find them in the year 1851; jumbling together abstract metaphysics or mental philosophy with organic psychology, and bewailing that we are "*hopelessly adrift* on the sea of conjecture about the truths of mental science."

We could point out to her half-a-dozen standard works, proving that we have a compass and a chart laid down to guide us safe among the shoals of metaphysical speculation on the one hand, and the rocks of scepticism or materialism, call it what we will, on the other. We may assure her that psychopathologists have already anticipated her queries, and that much of the learning of her coadjutor is after date. The only mode of forming true psychological deductions is, to compare the phenomena resulting from the normal state of the mental organ with the psychical changes consequent to morbid degeneration of its tissue.

"It remains," writes Mr. Atkinson, "for philosophers to place physiology and mental and moral philosophy in the same position as positive science reached by induction." Yes, we hope to do so, and had already commenced our course long before this solemn injunction; and if our Mentor will but contemplate the numberless establishments for the education of idiots, and the present enlightened system pursued in our asylums for the insane, he will learn how honestly we can vindicate the scientific philanthropy of our profession, and that he is not the first oracle in psychology, or the first great moralist born to teach mankind a new code of ethics.

He observes, very shrewdly, "There are not two philosophies,—one for mind and another for matter." To be sure not; where is the psychologist that has not been long aware of, and recorded, this truth? even without having dipped deep into the *novum organum* of Bacon. We have long come out of the "web of ideal creations," in which the bold sceptic asserts that we are still enmeshed.

We venerate the good parts of Bacon's character, and appreciate the indispensable value of the inductive philosophy. But it were surely somewhat uncourteous to blink altogether the wisdom of the present day—to be always looking back through the wrong end of the telescope of the mind, and quoting solitary apothegms and axioms of the sophs of former times, and becoming enamoured not only of their quaintness, but also of their very obscurity, instead of contemplating, studying, and analyzing modern psychological disquisitions which offer proof rather than speculation, and which *now* certainly disprove the affirmation of the "goodly Verulam," that "all the systems of the world are wrong, and founded in error."

The grand scope of this pair of intellectual lights, is to establish the doctrine of a universal law, a *self-existing, self-creating* law—a train of second causes without a first, which they term "nature." Here they would stop, denying altogether a Deity or a Providence. But they must have something more than negative assertion, before they determine this vital point; they must prove the origin of this self-existing law, a problem quite as difficult to solve as the origin of a Creator.

As philosophers, we must acknowledge and advocate the law on which science is altogether based; and we believe that "philosophy is not set in array against religion, when the student of nature endeavours to explain her phenomena by physical laws, for those laws the great Creator himself hath made."*

It is, however, by the recognition of this universal law that our male author is confident we shall "develop an universal love"—that is, to quote his own words—a state of liberty, equality, and fraternity—this felicitous consummation of Parisian glory having been hitherto thwarted by the writings of the fathers and the records of holy writ! We shall then, and not till then, penetrate the grand scheme of the universe. We shall perceive that "dirt is beauty unformed," that "evil is undeveloped good;" or, as Pope, forestalling Atkinson, has in a more devout spirit written—

> "All discord, harmony—not understood.
> All partial evil, universal good."

In the course of this deep volume the lady is the querist; but her own substantial opinions are very neatly and ingeniously dovetailed into her interrogatories, with something like that "darling sin" of Satan, "the pride that apes humility."

* Philosophy of Mystery.

It will be conjectured that the opinions are almost entirely of a very heterodox nature, not mincing the matter at all, but driving at once in *medias res.* Miss Martineau scouts altogether the "dignity" of man's origin, and expresses boldly her complete disbelief in the Mosaic records. And this, we think, on far more shallow grounds than those contained in the "Vestiges of Creation."

It is mere sophistry to urge recent geological discoveries in disproof of Holy Writ. When well studied, they often mutually confirm each other.

"The belief in the existence of a pre-adamite world presumes not to controvert the Mosaic record of the development of the globe, the creation of Adam, or the fall of man. Modern geology has peopled this pre-adamite world with saurians or lizards, a race of beings not concerned in the punishment of that delinquency. Of the existence of these creatures there is no doubt; the discovery of their fossil remains without a vestige of the human skeleton, marks the period of their destruction, and that the crust of the globe enveloping their relics, might have been reduced to that chaos when 'the earth was without form and void, and darkness was upon the face of the deep,' and from which our beautiful world was fashioned by a fiat." *

So that, granting the truth of our philosopher's estimation that "Mr. Lyell is a better authority than Moses," *we* see it all perfectly reconcilable with thirty thousand years' wear and tear of the waters of Niagara. The terms "chaos," "days" of creation, &c., &c., are equally coincident, if Mr. A. would candidly study them; but, as he himself writes, he will be "running on like an old gossip."

Although we fear it will be seen in the sequel that our feminine friend is tainted with a very consummate credulity—believing, in fact, in opposition to the aphorism of the Governor of Tilbury Fort, that she sees "what is not yet in sight"—yet nothing either sacred or profane will she take for granted which does not suit her purpose—she will not go a step beyond what she knows.

Now, what, we pray, would all science or history be reduced to, if we doubted and rejected all the records of our predecessors? Were we to do so, these would, indeed, be what Mr. Atkinson vain-gloriously affirms they *are*, "barbarous times!" But soft, the good time, the atheistic millennium, is coming, and then we shall see—ay, then we *shall* see. Then we shall believe nothing that does not tally with our own sight or touch. Pooh! we hear people say, Greenland was never green, because it is *now* white; that there never was any eruption of Vesuvius, because a solemn silence reigns at the mouth of the crater!

Miss Martineau's reasoning has prepared us for the hypothetical

* Philosophy of Mystery, p. 176.

aphorism—"Mind is the *product* of the brain—the manifestation or expression of the brain in action, as heat and light are of fire, and fragrance of the flower."

"Brain, however, is not more *identical* with mind than retina is with sight; but the mind cannot, of course, be *indicated* without brain, for, as the material world would be intact without a sense, so there can be no earthly *evidence* of mind without a brain, which may be termed the sense of the spirit."*

We are tired of the old analysis, so often adduced, of instruments and machines, the conception of man's mind and the work of man's hand, and set to work by man's ingenuity. In identifying the evolution of mind from the brain with that of heat and light from fire, the pseudo-phrenologist forgets that the caloric *pre*-existed, and was made sensible by the influence of oxygen. What is this caloric? not (to fight our philosopher with his own weapon) the *product* of a block of coal—it was there before as a property. That which, when developed, we term mind, was already in its organ. We must then go back beyond the manifestation of the product—the law—to that element, the product of which was effected *by* the law. The brain is affirmed to be a *gland, secreting mind* just as liver secretes the tangible and visible *bile,* the stomach the gastric juice. And from what are bile and gastric juice secreted?—blood, endued with vital properties. Deprive the liver and stomach of its blood, there will, of course, be no bile and gastric juice. Deprive the brain of its principle by a blow, although *it* may still have blood, and that vital and circulating, still there will be no mind. The influencing principle of the brain, therefore, even if it does secrete, differs essentially from that of gland, the productions of which are *palpable.*

Then, as to the organs of sense. The *globe of the eye* must *be* before there is sight, (we believe Miss Martineau would deny it,) as the brain must *be* before there is intellect. But as there must be light to give sight to the eye, there must be soul or something else to give intellect to the brain.

Now, in all this, the sceptic hugs himself that he and Spurzheim think alike; but the phrenologist does *not* affirm matter to be the only antecedent of mind, but that in it there is a special faculty adapted to our consciousness.

There must, therefore, be something beyond a law, which law *we* believe was created. It is folly to challenge us to prove its nature, or how it began. We cannot even conceive the nature of electricity, yet we *know* it to exist. We think the philosopher must explain the daily

* Philosophy of Mystery.

and multiform phenomena of our planet, ere he proudly presumes, not only to doubt, but to deny the perfection of "the great Spirit of the universe."

We are, however, taught by him a lesson in scepticism. He boasts himself, "regardless of the opinions of men." We will for once obey him, discard his, and keep to our own opinion. *He*, however, *does* worship one idol (of the quotations from whom there is no end)—Bacon—*toujours perdrix*—Bacon for ever! he exclaims; and yet one inference of Bacon tears up his material hypothesis by the roots. "The *tangible* parts of bodies are stupid things, and the spirits do in effect *all*." So, then, there *is* a spirit. Of a truth, the unanimity of Atkinson and Bacon is wonderful.

"Oh, if we could have Bacon back again!" also exclaims Harriet Martineau; yet we have observed, that with all their adoration, there is a *grana salis* of depreciation thrown in. This "meanest of mankind" is said by them to have been "ever practising the *craft* of his wit; and his religious professions were mere *shams!*"

To prove the peril also of basing an argument on the faith of a great name, he adduces his Magnus Apollo in proof of clairvoyance. "In the removing of cataract from the eyes, the little silver needle wherewith the cataracts are removed, even when it moveth upon the pupil within the coat of the eye, is excellently seen." Here are two errors: the needle is not silver; and not only the cornea, but the lens and humours refract and transmit.

We counsel Mr. Atkinson to divest his mind of the crotchet, that a metaphysician cannot reason from fact. Every moment of the study of the physiologist, the phrenophysiologist (we give him his tether), is, to use his own words, "observation of effects in relation to causes, in order to the discovery of the laws concerned."

One great stumbling-block to the fair discussion of the great psychical question has been the comparison of faculties—not to be compared—*instinct* and *reason*.

If reason be fairly analyzed, it will be found to be composed of certain qualities; *some*, indeed, of which, in different degrees, may be common to man and brute. Memory, distinguished essentially from recollection (which is voluntary), is the only one *highly* influential;—it is this which is the source of the myriads of zoological anecdotes so bewildering to the young psychologist.

The dog remembers the lash when he sees it, and even when he has done that for which he was whipped, by a low process of association, and he carries his tail between his legs; but he could not by an effort of the mind *recollect* until something occurred to *recal* it. The brute cannot from analogies look up from remote consequences to causes.

The law of peculiar instincts given to him impels him *periodically*, often blindly, to act where no reasonable motive exists. He does not follow up a continuous train of thought or record, or *think* of the thought as man does; although we may grant with Bacon that "there are some instances in the actions of brutes which seem to show that they too can syllogize;" yet differing, of course, from Bossuet, who, believing in the *anima brutorum*, promised the brute immortality.

The dog, *in solitude*, will not feel self-reproach or commit suicide as the human culprit; he will steal or kill, and yet dread nothing—but discovery. But man in a desert will reflect on his crime, and dread its penalty, though he has never seen his judge. The brute indulges his passions without control; man controls his propensities when religion, or virtue, or prudence forbid. This may be the result of the preponderance of brain over *medulla oblongata*.

Nor does the ingenuity of the brute naturally or progressively *improve;* the beaver built his wigwam, and the swallow her nest, in the same style when Pliny wrote as now.

It is this *high degree* of reason, which, with all their subtlety, our authors never arrogate in the brute, which makes man a thinker and a responsible being. Organization is the great theme of the materialist. We might meet him for a moment on his own ground, and ask him—As the brain of the sheep, of the elephant, and of man, so closely resemble each other, why, if organization be the *source* of mind, the manifestation is not resembling in all? Is it not a *necessity* that, in one, there must be something *superadded?*

The rejection of mere *craniology* is judicious; but now for the phrenological creed of Mr. Atkinson. He believes we have two brains. His duality is not, however, that of Wigan; it is cerebrum and cerebellum, or great and little, as every one knows. He assures us that only one side of the double cerebrum is in action at a time. How does he know this? Has he mesmerized the hemispherical ganglion for information, or has one of his clairvoyantes arrested a thought as it slunk away from a gland through the fibres of the tubular neurine?

Bigoted scepticism is as fatal to the cause of truth as blind faith. It is as perilous to say "this *cannot* be"—to give the dogmatical lie—as it is to bow slavishly to one authority—"*jurare in verba magistri.*" In the discussion of important questions, it is better to meet half-way—*there* we may often discover the truth. We do not believe a tithe of the vauntings of the clairvoyante, the excitement of special organs, the transference of senses, and remote and occult influences. We have ourselves detected too many impostures. But many of those influences, which seem to be novel or special, may be readily explained, on what Messrs. Atkinson and Martineau call *law,* on the principles of natural philosophy, without calling to our aid an *aura* or *blue fluid.*

We will even grant that there is a "sentience, independent of consciousness or will," when Mr. Atkinson feels an unusual sensation in passing his hand over an affected part. If there be increased temperature, and his hand be of lower degree of heat, it is a natural law that the unequal conditions should form a balance, and this transition must impart a peculiar sensation to a highly sensitive tissue. Such seems to be in an exaggerated degree the mystery of animal magnetism. So far we ought to grant, but the phenomenon is not new: friction, shampooing, electricity, even animal warmth, have been adopted as remedial means, long ere the ingenious Mesmer warped physiological truths to his own purpose. Even the exaltation of the senses is not an *abstraction* of animal magnetism; it occurs without the prestige of *passes.*

Here then we leave the mesmerist to soar, with his clairvoyantes, into the clouds of mystery, which (however his *amour propre* may blind him) he does infinitely beyond the most enthusiastic spiritualist.

But *we* must not invade the sacred domain of Miss Martineau's mesmeric seminary. It seems the specification sent in by Atkinson entitles him to a sort of patent privilege; for thus ingeniously does he oust the physician from the vestibule of the magnetic Eleusis: "Mesmerizing *doctors* have given diseases that they have brought from other houses to those whom they have mesmerized; and thus it may be a question if medical men are proper persons to mesmerize."

"When Bishop Berkeley said, 'there was no matter,'
And prov'd it,—'twas no matter what he said."

These material arguments are not material, say we, in explanation of those phenomena which the physician is daily witnessing.

It must be confessed, however, that there is something far more *real* in the maiden Martineau than in the matron Crowe: and we really prefer even the local phrenology of Atkinson to the psychical projectiles of her of the "night side," and to all other farragos of trash, with *ad captandum* titles, scratched off to gull the curiosity of the public. But is not our *couple* also guilty of *projectilism?* This wonderful partnership in heterodox philosophy may be but the substantial spirit of Mr. Atkinson projecting into that of Miss Martineau, for the doctrine of spiritual or ethereal influence at a distance, must be, according to their own showing, a fallacy.

The superstition of prophetic dream or trance—the prescience of events, *et id genus omne supernaturalium*, had been, we thought, long since explained, even up to the mark of a materialist's standard. We are, however, again overdosed with these modern miracles—some of them far eclipsing the wonders of Aubrey, and Glanville, and Moreton; and yet Mr. Atkinson startles us with this slight epitome of autobiography—"I do not think I am a very credulous man!" We do. The modesty of philosophy is exemplary. Witness also the following choice physiological *morçeau:* "We *know* that some can see distant objects without the *use* of the eye." What! a faculty without an organ! bile without liver! Is this chopping and changing of special functions strictly according to the unalterable and fixed LAWS of nature? Now, if this is not ultra-spiritualism, we do not understand plain English. To us it seems the most renegade apostasy and recantation we have lately met with.

Our sceptic has discovered a whole regiment of senses, sensations, faculties or feelings; nay, he makes every thought a sense. He tells us we can hear also through other parts of the body besides the ear. The undulations or vibrations of sound and the shooting of a ray, we thought, required a fluid and a lens in the internal ear and eye, ere the brain could be impressed; but physiological anatomy has, it seems, deceived us—it is not so. We could believe that in adducing the old hacknied experiment of the watch between the teeth, they supposed the teeth *heard* the sound, and we fear their readers *may*, some of them, believe so. The proximity of the Eustachian tube is entirely overlooked.

But there is no end in the volumes to these incongruities and false conclusions. "The sense of smell is said to exist without the olfactory nerve," and so on, *usque ad nauseam.*

According to the theory thus propounded, an organ is not essential to a function. Matter is *not* mind; there might be mind without brain in transcendental spiritualism!

The beautiful globe of the eye—its perfect lenses—its diaphanous humours — its fringed curtains — its most exquisite mechanism is made in vain—a useless appendage to the brain—fashioned merely to entangle men's hearts, and call forth the lover's rhapsody. The whole of its wondrous faculties can in a moment be displayed by the skin of the belly, if the Messrs. Atkinson do but project their souls or mesmeric energy into the body of a sensitive recipient.

How can we interpret this wise saw—"when the *ordinary* and outward action of the senses is cut off, and when the body is brought into a peculiar abnormal condition, the inner part of the brain might partake of the condition not required by the paralyzed senses." So he even believes that light is not essential to vision. When the eye is blinded it can see objects by reflex action! "*Credite Pisones.*" Harriet, of course, agrees implicitly with all this. "We have arrived," she writes, "at the greatest discovery ever made," &c.; and then she directly adds—"I have run on too long." For once we entirely coincide. A part of this great discovery is, that like magnetism, the fluid or aura leaps or is blown through space from one brain to another. This, of course, is one of those mysteries which "we must receive as fact," though "we cannot comprehend them." A door is thus open to *conjecture*:—and there is an end to that philosophy the "firm" pretend to teach.

Mr. Atkinson professes to "make mesmeric sleepers fancy that they have pain or pleasing sensations, or that they are in motion," &c.; rather a perilous freak this; let the lady who advertises to teach the power of making people fall in love with you at will, look to this. We have hitherto smiled—we must now frown at the profanation of reducing, not only natural magic, but even the miracles of Christ, to the mere result of animal magnetism. In the following blasphemy the cloven foot peeps out:—

"I was demesmerizing a patient, and the influence seemed to pass into a lady standing close by. The patient awoke, but the other ran screaming away like one possessed, and I thought of *the devils cast into the herd of swine.*"

We close the book on this sentence, in pity. Poor Swedenborg, too, the illustrious clairvoyant and high priest of the modern revelation, is laughed to scorn, while the deutero-scopia of the Martineau girls is gospel. Manuel is called a *madman* because he thought "his visions realities." If his revelation had been that of profane, instead of divine, mysteries, he would have been a *genius.*

Cupid, we allow, is very fair game, and his influence it seems consists in nothing but a mere vibration of the medullary chords of the organ of amativeness. "The note of one instrument sounding will cause a response from a corresponding note in another instrument. How similar is this to the sympathy which may be induced and which often spontaneously occurs in love, between two minds and bodies." Had they known this at the Agapemone, surely this vibratile recreation would have superseded the game of hockey.

Miss Martineau's free-thinking would often come out as the most unblushing infidelity, did she not shield herself behind the mask of a query. Her sixteenth letter is a tissue of interrogatory which would be a poser to any one but her *collaborateur.* "In speaking of God," she writes, "do you not use another name for law?" She need not have asked. But this harping on the subject of irresponsibility—annihilation—the self-existence of the law—and the creation of the Creator, are themes which we would fain waive and leave to the cognizance of the church or her Christian advocates, were they not so intimately associated with our especial studies.

For a more rational analysis of the causes of prophecy, dream, vision, the prophecies of Cazotte, of Joan of Arc, &c., we refer to a work to which we have already alluded.

The impious cosmogony of Heraclitus and Empedocles cannot be too severely censured, and when a modern philosopher of great power blazons them forth on his pages as a test, or illustration, we must not be satisfied with mere surmises and specious negations. We must even stain our pages with their quotation, to show the constant leaning to impiety, and the effrontery of these modern heathens:—

"The world was made neither by God or man; and it was, and is, and ever shall be, an ever-living fire in due measure self-enkindled, and in due measure self-extinguished."

"Wrongly do the Greeks suppose that aught begins or ceases to be; for nothing comes into being, nor is destroyed; but all is an aggregation or secretion of pre-existent things, so that all becoming might more correctly be called becoming-mixed, and all corruption, becoming-separate." Such texts as these, penned in the dark ages, are adduced, strange to say, as *arguments* by these modern philosophers, and their own, it will be seen, chime in admirably. "Philosophy finds no God in nature, nor sees the want of any." So, although every nation believes in a Deity, one Mr. Atkinson denies it, because philosophy does not find it! Therefore there *is* none. But where does he *find* his *law?* And how is it that the law has been so long in working out its mighty changes, which often display as great an interruption as a miracle? (A law should never vary.) And yet, till now, it has never discovered that "the forms of matter, and the condition of mind, which is one form of the properties of matter, are all determined by law, bound down by the adamantine chain of necessity."

We have heard a blasphemous whisper before on this awful point, and we remember, with something like horror, the death bed of the whisperer.

The solemn, the "terrible infinite," to which these infidels refer must be a deity or nothing. It is just as easy to conceive or believe in the existence of a Creator, as a creation; that a *divine power* has existed from all eternity, as that a world or a man have existed from all eternity.

And what is the upshot of all this fine reasoning? that the universe is a self-existent, and *purposeless,* because only a *temporary,* machine. Men are born only to breathe, and think, and act in ceaseless opposition, and then to die in agony, and be—nothing! And then Mr. Atkinson makes a virtue of this necessity, "Why should I require another life?"

We are really almost tired of this running fire, this battledore and shuttlecock game, for the coincidence of Martineau and Atkinson is a sort of argument in a circle. First, she agrees with him about the fallacy of a future estate, and the monomania of longing for it, or, as he says, "a pampered habit of mind."

So, in the end, because two or three bold people choose to disbelieve in futurity, the glorious minority directly stamp the myriads of believers as "drinkers or children:" modesty itself. Necessity tallies beautifully with this annihilated doctrine. "I cannot alter my will nor be other than what I am, and cannot deserve either reward or punishment." So if his will compels to murder he ought not to be punished. Lord Campbell, then, may shut up his court.

The mockery of the miracles is the old game of infidelity, and we have here a renewal of the blasphemy. Greatrex, Loutherborg, Aymar, and Atkinson himself, are vaunted as fully competent with their *mesmeric force* to perform them.

But to decry Scripture—to laugh those events to scorn which were foretold by many prophecies, when he himself is the great disciple of clairvoyance and *modern* prophecy, and has broached more barefaced hypotheses than any one we know of—is this not monstrous? He has, to use his own words, "set up reason in the judgment-seat," and has begun to preach, and ridicule, and jumble together, allegory and metaphor, history and scripture, without even explaining away one record of holy writ.

To one not in bondage of this sovereign reason—the design and scheme of the creation are clear. But it seems all kinds of phenomena are granted where there is no good result, no benefit conferred. Directly a moral purpose is the end, then out peeps a doubt or a blind contradiction; and an argument in favour of futurity or providence is at once, in Johnson's phrase, saluted with "You're a fool, and there's an end of it."

In broaching the theory of conception with the maiden lady, the quaintness and delicacy of this "fellow" are admirable. "What a chance," writes he, "is my existing at all. A minute later—nay, a second later, or the slightest change of circumstances in my conception, and it would not have been *I* that was born." He will surely make the lady "wise in her generation." And then he goes floundering on in a mist, regarding exigencies and development, which the physiologist could easily light him out of, but he pooh poohs them. If we assure him that the devil has been seen "flying out of a burning heretic in the shape of a flame of fire" he would smile in scorn, but when *he* assures us that "death appeared in the form of a black cat, on an old woman's bed," he exclaims, "*we must* believe."

And now comes the crowning heresy. "There are thousands of noble minds set free from the dogmas of Christianity, which they see to be neither reasonable nor moral. Christianity is not historically true," and thus runs on the strain of infidelity. Now, by what just rule this couple sets up one historical record against another, we know not. The *effects* of *pure* Christianity we have ever seen to be brotherly love and charity; but we are told in this book, "its result is, that the affections are perverted from their proper sphere of action, which is the love and companionship of their fellow-creatures." The grand injunction that shines throughout the code of Christianity is, "love one towards another." Yet Atkinson & Co. will have it, that it is only by the new birth, the love of nature, the living under the *law*, that men can "learn to forget themselves in the love of their fellow-men!"

But we must, as we professed, leave the divine to refute the infidel. For us to cite scriptural authority would be overstepping the limits of devout psychology. But the tissue of arrogance and blasphemy is so unblushing—the parallelism of Christ, for instance (whose "case," he says, "is as clear as daylight,") with Socrates and Swedenborg, and even with an American boy, named Davis, so gross—that we need but point to the odious page 212 to excite the pity and indignation of our readers.

It must be confessed that the heterodoxy of the spinster is gigantic. She has out-babeled Babel. Her city is built—her tower has reached Heaven. She is the great Titaness of the age. Philosophy, theology, religion, have been all hoodwinked for many thousand years, and Martineau and Atkinson have now torn off the mask.

Behmen, Santa Teresa, Swedenborg, merely presumed to *demonstrate* that heaven *was*—Martineau and Co. prove that heaven is *not*.

The question of responsibility is one of high importance—of almost unlimited extent in religion, morals, and jurisprudence.

But if man is irresponsible because he is impelled by necessity, will Atheism prevent crime? Oh, yes; witness the *prevised* effect of the atheistic millennium. "What repose begins to pervade the mind; what clearness of moral purpose naturally ensues; what a new perception of the beauty of holiness!"

Infidelity is, then, the purifying of the heart, irresponsibility the repose of the soul; since, as the spinster writes, "Christianity has not Christianized the world!"

Robert Owen once confessed to us that he must educate and bring up the universal mind to his own standard, ere his Utopian parallelograms could be established. So must we, if we hope to live safely under the LAW *of nature.* For Mr. Atkinson's ethnological classification is also rather a funny one. He says, our race is composed of lovers and poets, and of Shakspeare's madmen—the lunatic being the third. A hopeful world, truly, without religion's law to control that of nature—liberté, egalité, *without* fraternité. We see but little preparation for futurity; or, in Mr. Atkinson's words, "that enchanted life beyond the grave," under the protection of this atheistic *law.*

The sympathy of minds, or "thought-reading," is the great gun of the mesmerist. It is a notion more imaginative than aught that the spiritualist advances. The psychologist stops short of the Martineau spirituality. He believes mind in the present life can only be *evinced* through brain: *her* spirit-flights are far more ethereal a medium in a distance. *We* think thought is the peculiar province but not a *product* of the brain. Whether a cerebral vesicle is dislodged by each emotion we do not pause to discuss. But the mind in the estimation of these modern metaphysical luminaries, *is a principle capable of being transmitted from one brain to another, by an effort of the will!* We even have a sort of jesuitical confession, that all actions are through and by spirit-conditions. "Spirit acts on spirit and through spirit;" so, first, there must be an organ or matter, to produce action, or motion, or thought, and then spirit may be totally separate from matter, even in our present condition!

Again, it is said, "all your thoughts, and your whole condition, and those of *thousands of others,* may be lying latent in *my* constitution at this moment." A most capacious receptacle. Truly Mr. Atkinson's brain is receiver-general. Thought, then, divides and multiplies itself, and passes, by stealth, into another's brain! This is their theory of dreams, spectres, and dying visions. Glyndwr, hide your diminished head! His calling spirits from the vasty deep was fudge—Harriet Martineau can call them from the brain, and they *come* at her call. Indeed, everything is at her beck. Even common cold obeys Atkinson's will, if he do but gently approximate to the invalid. Even the *material* influence of the dying flies off and infects the bystanders. It seems that "force is *realized.*" But is it not, again, rather paradoxical, that when the brain is weakening (sense and thought being *born of it*), the senses should be more acute?

But the Martineau code does not stop short in cold philosophy. It is to be the principal element in the grand fountain of virtue in the mesmeric millennium; "the knowledge which mesmerism gives of the influence of body on body, will bring about a *morality* we have not yet dreamed of."

We considered body as a machine, fraught with instincts and passions, which would run wild if not under the control of devotion; but as two negatives are said to make an affirmative, so two vices we suppose make a virtue.

Miss Martineau will no doubt complain that we have dealt severely with her. But if she will think of the proper answer to her own question, "Whose profession is it to observe the laws of man's nature and development?" she must confess, it is that of the psycho-physiologist. It is his constant duty to study deeply these laws in health and disease; the only mode by which any knowledge of legitimate psychology, or the blending of mind and body, can be attained.

After very close consideration, we must inveigh against the perilous tendency of this dissertation on the minds of those who are captivated by novelty and opposition, and a learned and acute phraseology. Our authors are determined to disbelieve, but their *real* opinions perhaps are more in accordance with physio-psychology than they themselves are aware of. To call God *nature,* to call physiology, in its wide meaning, *law,* is but to substitute other names in the place of those fully recognised; their infidelity consists in the blind denial of a *providence,* a ruler of the universe.

To say, man is the result of organization, is merely saying that organization is man.

But even their errors and their undevout reasonings are often expressed with an apparent power and acuteness. Yet there is little merit in this. It is the immense advantage of opposition and scepticism, to catch the ear of mankind by bold and novel language. *Mens hominum novitatis avida est.* With this concession we have fulfilled our duty in expressing our most decided disapproval of this book. And the authors will at once understand, that if their will be their law —if *they* are impelled by necessity to disbelieve, *we* are in like manner impelled to censure and condemn.

